# Troglitazone Impedes the Oligomerization of Sodium Taurocholate Cotransporting Polypeptide and Entry of Hepatitis B Virus Into Hepatocytes

**DOI:** 10.3389/fmicb.2018.03257

**Published:** 2019-01-08

**Authors:** Kento Fukano, Senko Tsukuda, Mizuki Oshima, Ryosuke Suzuki, Hideki Aizaki, Mio Ohki, Sam-Yong Park, Masamichi Muramatsu, Takaji Wakita, Camille Sureau, Yuki Ogasawara, Koichi Watashi

**Affiliations:** ^1^Department of Virology II, National Institute of Infectious Diseases, Tokyo, Japan; ^2^Department of Analytical Biochemistry, Meiji Pharmaceutical University, Kiyose, Japan; ^3^Liver Cancer Prevention Research Unit, Center for Integrative Medical Sciences, RIKEN, Wako, Japan; ^4^Department of Applied Biological Science, Tokyo University of Science, Noda, Japan; ^5^Drug Design Laboratory, Graduate School of Medical Life Science, Yokohama City University, Yokohama, Japan; ^6^Laboratoire de Virologie Moléculaire, Institut National de la Transfusion Sanguine, CNRS, INSERM U1134, Paris, France; ^7^JST CREST, Saitama, Japan

**Keywords:** HBV, internalization, NTCP, oligomerization, multimerization, troglitazone, entry, preS1

## Abstract

Current anti-hepatitis B virus (HBV) agents, which include nucleos(t)ide analogs and interferons, can significantly suppress HBV infection. However, there are limitations in the therapeutic efficacy of these agents, indicating the need to develop anti-HBV agents with different modes of action. In this study, through a functional cell-based chemical screening, we found that a thiazolidinedione, troglitazone, inhibits HBV infection independently of the compound's ligand activity for peroxisome proliferator-activated receptor γ (PPARγ). Analog analysis suggested chemical moiety required for the anti-HBV activity and identified ciglitazone as an analog having higher anti-HBV potency. Whereas, most of the reported HBV entry inhibitors target viral attachment to the cell surface, troglitazone blocked a process subsequent to viral attachment, i.e., internalization of HBV preS1 and its receptor, sodium taurocholate cotransporting polypeptide (NTCP). We also found that NTCP was markedly oligomerized in the presence of HBV preS1, but such NTCP oligomerization was abrogated by treatment with troglitazone, but not with pioglitazone, correlating with inhibition activity to viral internalization. Also, competitive peptides that blocked NTCP oligomerization impeded viral internalization and infection. This work represents the first report identifying small molecules and peptides that specifically inhibit the internalization of HBV. This study is also significant in proposing a possible role for NTCP oligomerization in viral entry, which will shed a light on a new aspect of the cellular mechanisms regulating HBV infection.

## Introduction

Hepatitis B virus (HBV) infection represents a major public health challenge; worldwide, approximately 240 million individuals are carriers, and chronic infection is a major risk factor for the development of liver cirrhosis and hepatocellular carcinoma (Ott et al., 2012; Zeisel et al., 2015). Current anti-HBV therapies include nucleos(t)ide analogs and interferons (IFNs) (Liang et al., 2015; Levrero et al., 2018). Nucleos(t)ide analogs, including lamivudine, adefovir, entecavir, telbivudine, and tenofovir, suppress HBV replication through inhibition of viral reverse transcription. IFNα and its pegylated form modulate the host immune response to HBV infection and/or directly inhibit viral replication in hepatocytes. These antiviral agents can significantly reduce the viral load in patients and induce remission of hepatitis; however, long-term treatment with some of these nucleos(t)ide analogs can select drug-resistant viruses, thereby decreasing therapeutic efficacy. Additionally, IFNs can present severe adverse effects with low tolerability. Moreover, complete elimination of HBV from infected cells is still a challenge, even with these anti-HBV agents. To achieve a cure, the development of new anti-HBV agents with different modes of action is needed.

Viral entry, a process that is critical for the initiation, spread, and maintenance of infection, is an attractive target for the development of antiviral agents (Testoni et al., 2017; Tu and Urban, 2018). HBV enters host hepatocytes through a multi-step process that is initiated by low-affinity viral attachment to host hepatocytes (Sureau and Salisse, 2013); this attachment is followed by specific and high-affinity interaction of the HBV large surface protein (LHBs) with an entry receptor, sodium taurocholate cotransporting polypeptide (NTCP), via the preS1 region of LHBs (Schulze et al., 2007; Yan et al., 2012). The preS1-NTCP interaction appears to trigger internalization by an as-yet unknown mechanism (Karayiannis, 2017). To date, a series of HBV entry inhibitors has been reported, including a lipopeptide (Myrcludex-B), bile acids, FDA-approved drugs (cyclosporin A, irbesartan, ezetimibe, and rapamycin), and additional compounds (including vanitaracins, proanthocyanidin, SCY995, NPD8716, and WL4; Lucifora et al., 2013; Blanchet et al., 2014; Iwamoto et al., 2014; König et al., 2014; Ni et al., 2014; Nkongolo et al., 2014; Watashi et al., 2014; Yan et al., 2014; Kaneko et al., 2015, 2018; Ko et al., 2015; Tsukuda et al., 2015, 2017; Veloso Alves Pereira et al., 2015; Wang et al., 2015; Donkers et al., 2017; Shimura et al., 2017; Passioura et al., 2018; Saso et al., 2018; Song et al., 2018). These compounds target the host NTCP protein or HBV particles themselves, inhibiting the attachment of HBV to the host cell surface. In contrast, no HBV-specific inhibitor has been reported to inhibit the viral internalization process. Moreover, the molecular mechanisms regulating HBV-NTCP internalization remain poorly characterized.

In this study, we identified troglitazone as an HBV entry inhibitor with a novel mode of action. Troglitazone did not inhibit HBV attachment but instead interrupted HBV preS1-NTCP internalization. We also showed that troglitazone disrupted the oligomerization of NTCP, suggesting a possible role for NTCP oligomerization in HBV-NTCP internalization. This hypothesis was further supported by the demonstration that peptides that competitively disrupted NTCP oligomerization also interrupted HBV preS1-NTCP internalization. Thus, our study provides the first example of agents that specifically inhibit HBV internalization.

## Materials and Methods

### Reagents

Troglitazone was purchased from AdooQ BioScience. Ciglitazone, mitoglitazone, and trolox were purchased from Cayman Chemical. Rosiglitazone, pioglitazone, darglitazone, netoglitazone, and SR-202 were purchased from Sigma-Aldrich. Balaglitazone was purchased from MedChemExpress. Inolitazone was purchased from ChemScene. Bafilomycin A1 was purchased from Wako. A preS1 peptide consisting of aa 2-48 of the HBV preS1 region with amino-terminal myristoylation, as well as carboxy-terminal 6-carboxytetramethylrhodamine (TAMRA)-conjugated derivatives thereof, were synthesized by Scrum, Inc. The NTCP peptide fragment library was synthesized by Sigma-Aldrich. Entecavir was purchased from Santa Cruz Biotechnology.

### Plasmid Construction

Expression plasmid, pEF4-NTCP-myc-His was constructed as described previously (Shimura et al., 2017). A fragment encoding hemagglutinin (HA)-tagged hNTCP was amplified using pEF4-NTCP-myc-His as a template with the primer pair 5′-CGCTACCGGTCTCGAGATGGAGGCCCACAACGCGTCT-3′ and 5′-CTGCAGAATTCTCGAGCTAAGCGTAATCTGGAACATCGTATGGGTAGGCTGTGCAAGGGGAGCAGTC-3′. The resulting amplicon was digested with *Xho*I and ligated into similarly digested pCSII-EF-MCS, yielding plasmid pCS-EF-hNTCP-HA (kindly provided by Dr. Hiroyuki Miyoshi at RIKEN BioResource Research Center).

### Cell Culture

HepG2-hNTCP-C4, Hep38.7-Tet, HepG2.2.15.7, HepG2, 293T, Huh-7, and Huh-7.5.1 cells, and primary human hepatocytes (PhoenixBio Co., Ltd.) were cultured in the respective cell culture media (e.g., the media composed of DMEM/F-12 + GlutaMax (Thermo Fisher Scientific) supplemented with 10 mM HEPES, 100 units/mL penicillin, 100 μg/mL streptomycin, 10% FBS, and 5 μg/mL insulin was used for culturing HepG2 and its derivative cells) at 37°C, 5% CO_2_, as described previously (Iwamoto et al., 2014; Ogura et al., 2014; Ishida et al., 2015; Nakajima et al., 2016). Huh-7.5.1 cells were kindly provided by Dr. Francis Chisari at The Scripps Research Institute.

### HBV Preparation and Infection

**Figure 1:**
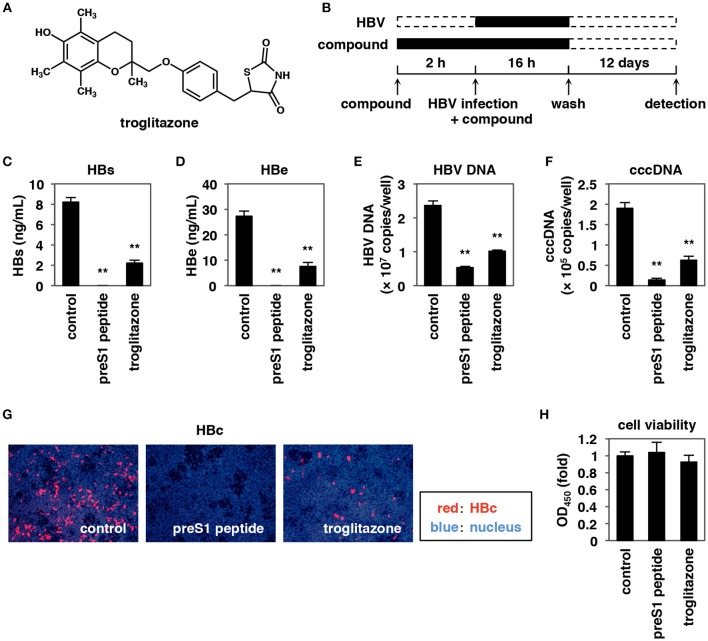
Troglitazone inhibits HBV infection. **(A)** Chemical structure of troglitazone. **(B)** Schematic representation of the protocol for treating HepG2-hNTCP-C4 cells with compounds and HBV. HepG2-hNTCP-C4 cells were pretreated with compounds for 2 h and then were inoculated with HBV in the presence or absence of compounds for 16 h. After the washing out of free HBV and compounds, the cells were cultured in the absence of compounds for an additional 12 days, and HBV infection was evaluated by quantifying each of the markers shown in **(C–G)**. Black and dotted boxes indicate the periods with and without treatment, respectively. **(C–H)** HepG2-hNTCP-C4 cells treated with or without 100 nM preS1 peptide or 25 μM troglitazone were inoculated with HBV according to the protocol shown in **(B)**. HBV infection was examined by ELISA detection of HBs **(C)** and HBe **(D)** antigens in the culture supernatant, by real-time PCR detection of HBV DNA **(E)** and cccDNA **(F)** in the cells, and by immunofluorescence analyses to detect HBc antigen **(G)** in the cells. Cell viability was also measured by the MTT assay **(H)**. Red and blue signals in **(G)** indicate HBc antigen and the nucleus, respectively. Data are shown as mean ± SD. Statistical significance was determined using a two-tailed non-paired Student's *t*-test (^**^P < 0.01).

**Figure 2:**
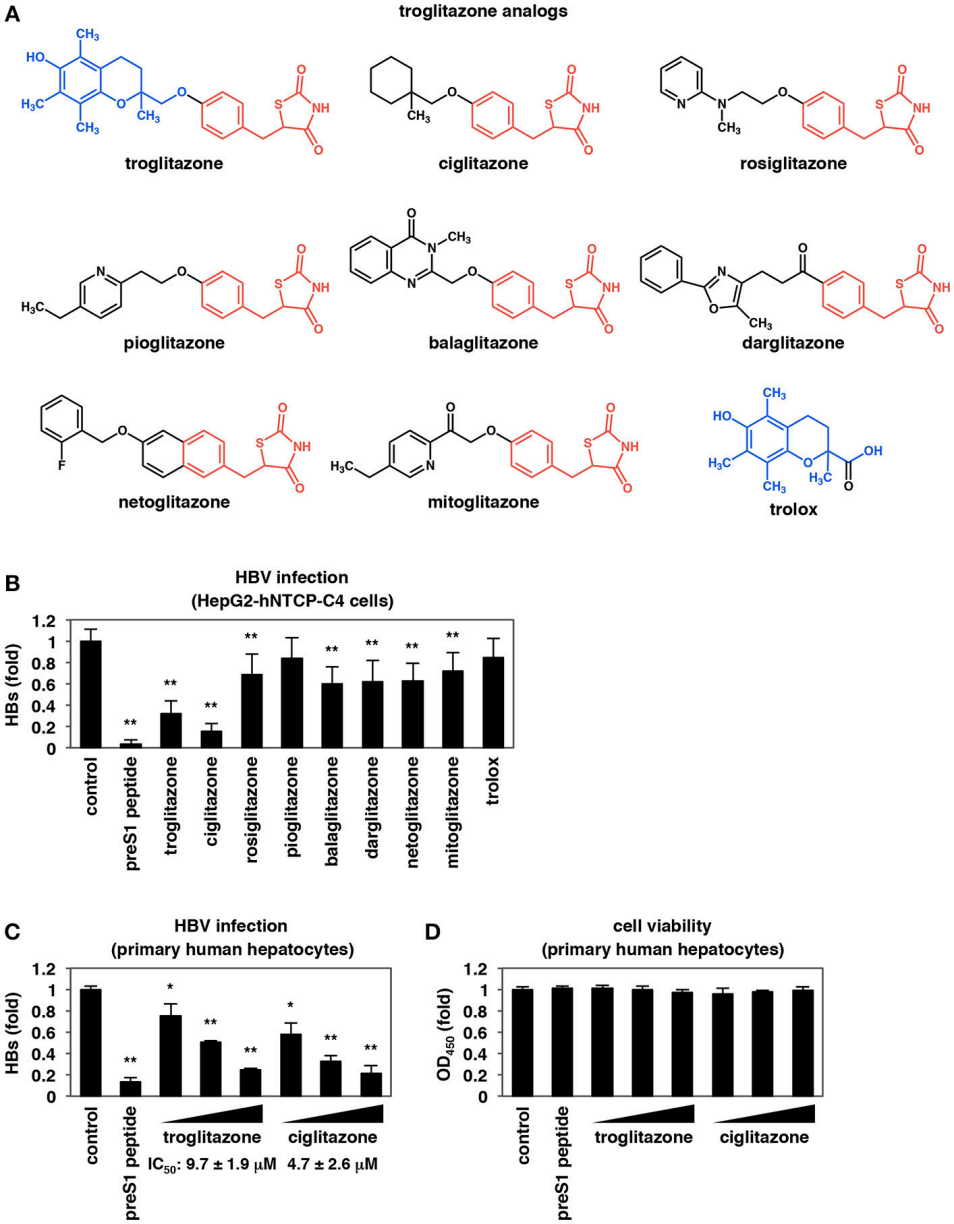
Analysis of troglitazone analogs. **(A)** Chemical structures of troglitazone analogs. The structures shared in common among troglitazone and its analogs are shown by red and blue. **(B)** HepG2-hNTCP-C4 cells were inoculated with HBV following treatment with 100 nM preS1 peptide or 25 μM troglitazone analogs as shown in Figure 1B. HBV infection was evaluated by quantifying HBs antigen in the culture supernatant. **(C,D)** Primary human hepatocytes were treated with or without the indicated compounds (100 nM preS1 peptide and 3, 10, and 30 μM of troglitazone or ciglitazone) and inoculated with HBV as shown in Figure 1B. HBs antigen **(C)** secreted into the culture supernatant was detected by ELISA. Cell viability was also measured by the MTT assay **(D)**. Data are shown as mean ± SD. Statistical significance was determined using a two-tailed non-paired Student's *t*-test (^*^P < 0.05, ^**^P < 0.01).

**Figure 3:**
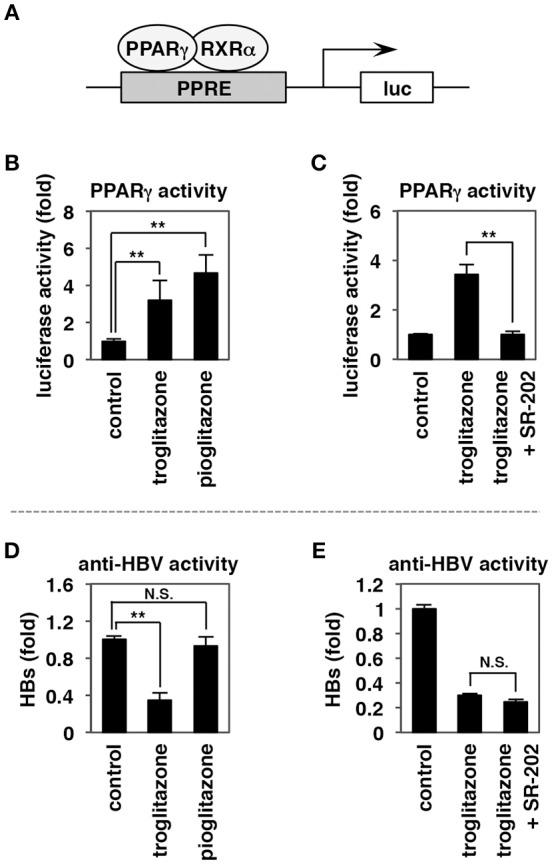
The anti-HBV activity of troglitazone is not mediated via PPARγ activity. **(A–C)** HepG2-hNTCP-C4 cells were transfected with a reporter plasmid carrying binding elements for PPAR (PPRE) together with expression plasmids encoding PPARγ and RXRα **(A)**. The cells were treated with or without 25 μM troglitazone, 25 μM pioglitazone **(B)**, or 25 μM troglitazone in the presence or absence of 1 mM SR-202, a PPARγ antagonist, for 24 h **(C)**, and luciferase reporter activity was measured. **(D,E)** HepG2-hNTCP-C4 cells treated with or without 25 μM troglitazone, 25 μM pioglitazone **(D)**, or 25 μM troglitazone in the presence or absence of 1 mM SR-202 **(E)** were subjected to the HBV infection assay according to the scheme shown in Figure 1B. HBV infection was evaluated by quantifying HBs antigen in the culture supernatant. Data are shown as mean ± SD. Statistical significance was determined using a two-tailed non-paired Student's *t*-test (N.S.; not significant, ^**^P < 0.01).

**Figure 4:**
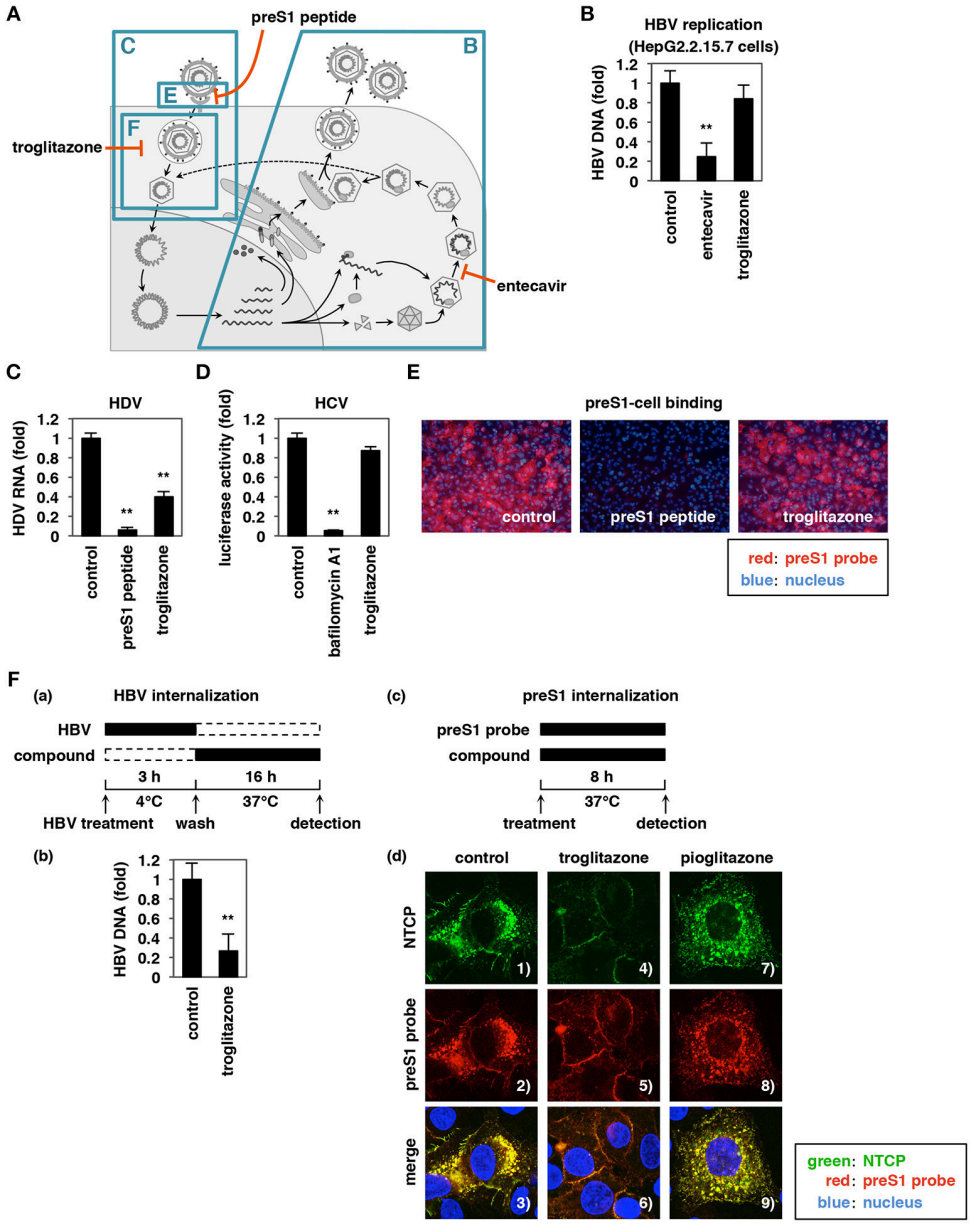
Troglitazone inhibits HBV internalization. **(A)** Schematic representation of the HBV life cycle. The blue boxes show the life cycle processes that were examined in the experiments in **(B,C,E,F)**. **(B)** HBV replication was evaluated by quantifying HBV DNA in HepG2.2.15.7 cells treated for 6 days with or without 1 μM entecavir (as a positive control) or 25 μM troglitazone. **(C)** HDV infection assay. HepG2-hNTCP-C4 cells were inoculated with HDV in the presence or absence of 100 nM preS1 peptide or 25 μM troglitazone, according to the protocol shown in Materials and Methods. HDV infection was evaluated by quantifying HDV RNA in the cells by real-time RT-PCR. **(D)** HCV infection was evaluated using the HCV pseudoparticle system, as described in Materials and Methods, following treatment with or without 10 nM bafilomycin A1 as a positive control or 25 μM troglitazone. **(E)** HBV preS1-mediated attachment to host cells was examined in HepG2-hNTCP-C4 cells exposed for 30 min at 37°C to TAMRA-labeled preS1 peptide (preS1 probe) in the presence or absence of 100 nM non-labeled preS1 peptide or 25 μM troglitazone. Red and blue signals indicate preS1 probe and the nucleus, respectively. (**Fa**) Schematic of the experimental protocol used for examining HBV internalization. HepG2-hNTCP-C4 cells were treated with HBV at 4°C for 3 h to allow HBV-cell attachment without internalization into the cells. After free HBV was washed out, the cells were cultured for 16 h at 37°C in the presence or absence of 25 μM troglitazone to allow viral internalization. The cells then were trypsinized and extensively washed to remove the cell surface HBV, and HBV DNA in the cells was quantified **(b)**. **(c)** Schematic of the experimental protocol used for examining preS1 internalization. HepG2-hNTCP-C4 cells were exposed for 8 h at 37°C to preS1 probe (red) in the presence or absence of 25 μM troglitazone or pioglitazone, and then were stained for NTCP (green) and the nucleus (blue) by immunofluorescence analysis **(d)**. Merged patterns are shown in the bottom panels. Data are shown as mean ± SD. Statistical significance was determined using a two-tailed non-paired Student's *t*-test (^**^P < 0.01).

HBV in all the experiments was handled in a biosafety level 2 laboratory by following the national and institutional rules and regulations. HBV derived from the culture supernatant of Hep38.7-Tet cells prepared as described previously (Watashi et al., 2014) was used as the HBV inoculum. HBV was inoculated at 12,000 (Figures 1, 2B, 3D,E, 4F, **6Ac** and Figure S6) or 500 (Figure 2C) genome equivalents (GEq) per cell in the presence of 4% polyethylene glycol 8000 (PEG 8000; Sigma-Aldrich, P2139) at 37°C for 16 h, as described previously (Watashi et al., 2013).

### Detection of HBs and HBe Antigens

HBs and HBe antigens were quantified by enzyme-linked immunosorbent assay (ELISA) using anti-HBs (LifeSpan BioSciences) or anti-HBe (LifeSpan BioSciences) antibodies essentially as described previously (Passioura et al., 2018). The half-maximal inhibitory concentration (IC_50_) value of the compounds was calculated with the linear regression of the dose-response curve for compound concentration against HBs level in a log-log graph.

### Real-Time PCR

Real-time PCR for quantification of HBV DNA and covalently closed circular DNA (cccDNA) was performed with primers and TaqMan probe as described previously (Passioura et al., 2018).

### MTT Assay

The 3-(4, 5-dimethylthial-2-yl)-2, 5-diphenyltetrazolium bromide (MTT) assay for measurement of cell viability was performed as described previously (Passioura et al., 2018).

### Indirect Immunofluorescence Analysis

Immunofluorescence analysis was performed as described previously using an anti-HBc antibody (Thermo Fisher Scientific, RB-1413-A) at a dilution of 1:100 (Passioura et al., 2018).

### Reporter Assay

HepG2-hNTCP-C4 cells were transfected with a reporter plasmid carrying a tandem repeat of the peroxisome proliferator-activated receptor (PPAR) response elements (PPREs) upstream of the firefly luciferase gene, together with expression plasmids encoding PPARγ and retinoid X receptor (RXR) α, using TransIT-LT1 (Takara Bio Inc.) according to the manufacturer's protocol. The cells were treated with the indicated compounds for 24 h and the luciferase activity was measured as described (Watashi et al., 2013) to assess the transcriptional activity of PPARγ.

### HBV Replication Assay

Starting at 3 days post-seeding, HepG2.2.15.7 cells were treated with compounds for 6 days. To measure HBV replication, HBV DNA released into the culture supernatant was quantified by real-time PCR, as described previously (Shimura et al., 2017).

### Hepatitis D Virus (HDV) Infection Assay

HDV was prepared from the culture supernatant of Huh-7 cells transfected with pSVLD3 (a kind gift from Dr. John Taylor at the Fox Chase Cancer Center) and pT7HB2.7 plasmids, as described previously (Kuo et al., 1989; Sureau et al., 1994; Gudima et al., 2007). HDV was inoculated to HepG2-hNTCP-C4 cells at 15 GEq per cell in the presence of 5% PEG 8000 and the mixture was incubated at 37°C for 16 h, as described previously (Kaneko et al., 2015).

### Hepatitis C Virus (HCV) Pseudoparticle Assay

HCV envelope-dependent entry was examined using the HCV pseudoparticle system. HCV pseudoparticles were prepared with the culture supernatant of 293T cells transfected with expression plasmids encoding the HCV E1E2, murine leukemia virus Gag-Pol, and luciferase (kindly provided by Dr. Francois-Loic Cosset at the Universite de Lyon) as described previously (Bartosch et al., 2003). Huh-7.5.1 cells preincubated with compounds for 1 h were inoculated with HCV pseudoparticles in the presence of compounds and the mixture was incubated for 4 h. After the washing out of virus and compounds, cells were incubated for an additional 72 h and then lysed to measure luciferase activity (Nakajima et al., 2013).

### PreS1 Binding Assay

PreS1-cell surface attachment was evaluated by incubating the cells at 37°C for 30 min in the presence of TAMRA-labeled peptide spanning aa 2–48 of the myristoylated preS1 region (also referred to as preS1 probe), as described previously (Kaneko et al., 2015).

### HBV Internalization Assay

HepG2-hNTCP-C4 cells were treated with HBV at 4°C for 3 h to allow HBV to attach to the cells without endocytosis. After the washing out of free HBV with the cell culture media, the cells were cultured at 37°C for 16 h to allow virus internalization. The cells then were trypsinized and extensively washed prior to quantifying the level of intracellular HBV DNA (Watashi et al., 2014).

### PreS1 Internalization Assay

HepG2-hNTCP-C4 cells were exposed to preS1 probe at 37°C for 8 h to allow the attachment and internalization of the preS1 probe. The cells then were washed, fixed, and treated sequentially with anti-NTCP antibody (primary antibody; diluted 1:20) and Alexa 488-conjugated goat anti-mouse immunoglobulin G (H+L) (secondary antibody; diluted 1:1,000) to visualize NTCP. The nucleus was counter-stained with 4, 6-diamidino-2-phenylindole (DAPI) at a dilution of 1:5,000.

### Proximity Ligation Assay (PLA)

NTCP oligomerization was evaluated using PLA (Sigma-Aldrich) in cells transfected with expression plasmids for both myc-tagged NTCP and HA-tagged NTCP and treated with preS1 probe. PLA signal produced using anti-myc (Santa Cruz Biotechnology, A-14) and anti-HA (Abcam, HA-7) antibodies was detected according to the manufacturer's protocol.

### Pull-Down Assay

**Figure 5:**
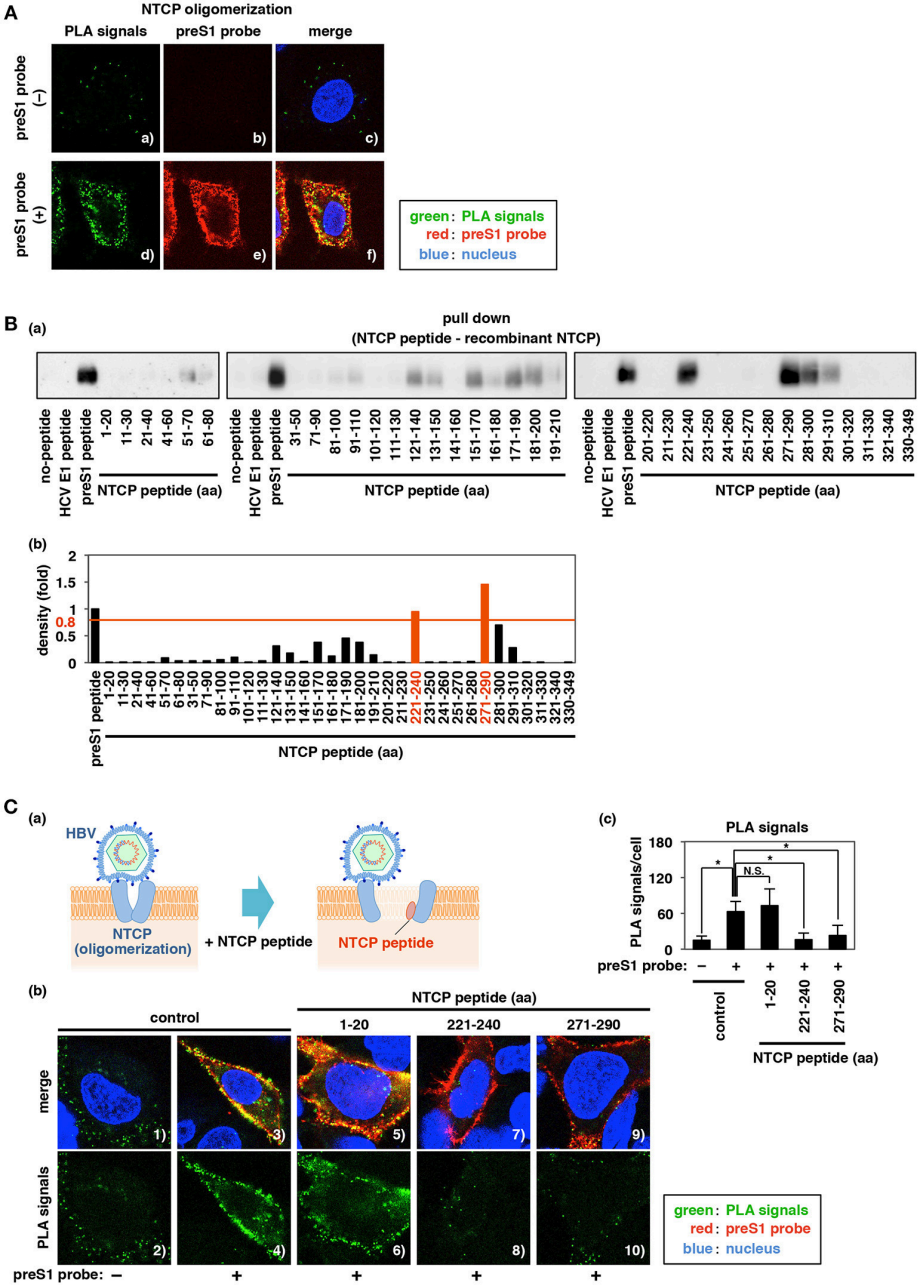
Identification of peptides interacting with NTCP and impeding NTCP oligomerization. **(A)** Proximity ligation assay (PLA) to evaluate NTCP oligomerization. HepG2 cells overexpressing both myc-NTCP and HA-NTCP were treated for 30 min at 4°C with or without preS1 probe (red), and then the PLA signal (green) produced by the proximity of anti-myc and anti-HA antibodies was detected with Duolink PLA. The nucleus was also stained with DAPI (blue). Merged images of green, red, and blue signals are provided in the right panels. **(B)** Interaction between recombinant full-length His-tagged NTCP protein (His-NTCP) and the fragment peptides derived from NTCP, as examined by pull-down assay. Biotinylated peptides of HCV E1 (aa 111–140), preS1 (aa 2–48), or each of 34 NTCP fragments (20-aa lengths corresponding to the indicated regions) immobilized on streptavidin-agarose beads were incubated with His-NTCP. Co-immunoprecipitated His-NTCP was detected with anti-His antibody by immunoblot **(a)**. Quantitative densitometry is shown in **(b)**; values for the co-precipitated His-NTCP bands were normalized to that obtained with the preS1 peptide (defined as 1.0). **(C)** The effect of NTCP peptides (aa 1–20, aa 221–240, and aa 271–290) on NTCP oligomerization was observed using PLA, as shown in **(A)**. PLA signal was detected in HepG2 cells overexpressing myc-NTCP and HA-NTCP following treatment with or without the indicated NTCP fragment peptides either in the presence (panels 3–10) or absence (panels 1–2) of preS1. **(b)** The lower panels show the PLA signal (green) only, and the upper panels show the merged images of green (PLA signal), red (preS1 probe), and blue (nucleus) signals. **(c)** PLA signals in the experiment in **(b)** were quantified using Dynamic Cell Count (KEYENCE). Data are shown as mean ± SD. Statistical significance was determined using a two-tailed non-paired Student's *t*-test (N.S.; not significant, ^*^P < 0.05).

Recombinant full-length NTCP protein tagged with His (His-NTCP) was incubated at 4°C for 4 h with either biotinylated NTCP peptide fragments (corresponding to 20-aa lengths of the NTCP sequence), preS1 peptide (as a positive control), or HCV E1 peptide (as a negative control). Biotinylated peptides then were “pulled down” with streptavidin beads and the resulting precipitates were dissolved in sample buffer. Coprecipitated recombinant His-NTCP protein was detected by immunoblot analysis with anti-His antibody (Tsukuda et al., 2017). The raw data (i.e., full image of the original gel) for the values presented in Figure 5Ba are shown in Figure S1.

### Statistics

Statistical significance was determined using a two-tailed non-paired Student's *t*-test. *P*-values < 0.05 were considered significant. Where applicable, *P*-values are indicated as ^*^P < 0.05 or ^**^P < 0.01.

## Results

### Troglitazone Inhibits HBV Infection

We employed a cell-based chemical screen using HBV-susceptible HepaRG cells (Gripon et al., 2002; Watashi et al., 2014) to identify small molecules capable of decreasing HBV infection. In this screen, troglitazone (5-[[4-[(3,4-dihydro-6-hydroxy-2,5,7,8-tetramethyl-2H-1-benzopyran-2-yl)methoxy]phenyl]methyl]-2,4-thiazolidinedione) (Figure 1A) showed strong anti-HBV activity. The anti-HBV activity of troglitazone was evaluated by an HBV infection assay using HepG2-hNTCP-C4 cells, a HepG2-derived cell line engineered to overexpress human NTCP (Iwamoto et al., 2014). HepG2-hNTCP-C4 cells were pretreated with compounds for 2 h and then inoculated with HBV in the presence of compounds for another 16 h. After the washing out of free HBV and compounds, the cells were cultured for an additional 12 days in the absence of compounds, as shown in Figure 1B. HBV infection was evaluated by measuring the levels of HBs and HBe antigens in the culture supernatant as well as the intracellular levels of HBV DNA, covalently closed circular DNA (cccDNA), and HBc antigen. PreS1 peptide, a lipopeptide consisting of myristoylated aa 2–48 of the preS1 region, a known HBV entry inhibitor (Gripon et al., 2005), was used as a positive control; in the presence of preS1 peptide, each of the tested markers of HBV infection were remarkably reduced (Figures 1C–G). Treatment with troglitazone obviously decreased the levels of HBs (Figure 1C) and HBe (Figure 1D) antigens, HBV DNA (Figure 1E), cccDNA (Figure 1F), and HBc antigen (Figure 1G) without significant cytotoxic effects (Figure 1H). These data suggested that troglitazone inhibits HBV infection.

### Identification of a Troglitazone Analog With Higher Anti-HBV Potency

We next examined a series of analogs to define a structure-activity relationship and to identify compounds having higher anti-HBV potency than troglitazone. As troglitazone consists of thiazolidinedione (Figure 2A, red) and chromanol moieties (Figure 2A, blue), we tested two series of analogs (Figure 2A): those carrying the thiazolidinedione moiety (Figure 2A, red) and those carrying the chromanol moiety (Figure 2A, blue). As shown in Figure 2B, most of the compounds carrying the thiazolidinedione moiety, except for pioglitazone, showed significant reductions in HBV infection, while trolox (which is almost identical to the chromanol moiety of troglitazone) had no anti-HBV effect. Although many of the thiazolidinedione compounds had lower anti-HBV activity than troglitazone, ciglitazone showed the strongest inhibitory activity against HBV infection (Figure 2B). This analog analysis suggested that the thiazolidinedione moiety, rather than the chromanol moiety, is necessary (but not sufficient) for the inhibition of HBV infection. This analysis further suggested that modification with (1-methylcyclohexyl)methoxyl, a side chain that is shared by troglitazone and ciglitazone, increases the anti-HBV effect. In contrast, thiazolidinedione compounds, which do not have any branched side chain from the main chain with relatively smaller molecular size (e.g., pioglitazone), showed little or no effect on HBV infection. To quantify the anti-HBV activities of troglitazone and ciglitazone, we performed the HBV infection assay in primary human hepatocytes treated with different concentrations of the two reagents. Both compounds showed a dose-dependent reductions in HBV infection (Figure 2C) without significant cytotoxicity (Figure 2D). The half-maximal inhibitory concentrations (IC_50_s) of troglitazone and ciglitazone were 9.7 ± 1.9 and 4.7 ± 2.6 μM, respectively. Thus, ciglitazone was shown to have higher anti-HBV activity than troglitazone.

### PPARγ Activity Is Not Responsible for Anti-HBV Activity

Compounds carrying thiazolidinedione structure (Figure 2A, red; such as troglitazone and pioglitazone) are well-known as agonists for peroxisome proliferator-activated receptor γ (PPARγ), a member of the nuclear hormone receptor superfamily involved in adipogenesis, lipid metabolism, inflammation, and maintenance of metabolic homeostasis (Lehmann et al., 1995). To address whether PPARγ activation is responsible for the observed inhibition of HBV infection, we used the thiazolidinedione compounds to treat HepG2-hNTCP-C4 cells transfected with both a reporter plasmid carrying the PPAR response elements (PPREs) upstream of a luciferase gene and expression plasmids for PPARγ and its heterodimer partner, retinoid X receptor (RXR) α (Figure 3A). Although activation of PPARγ-mediated transcription was observed following treatment with either troglitazone or pioglitazone (Figure 3B), significant inhibition of HBV infection was observed only with troglitazone, and not with pioglitazone (Figure 3D). Moreover, the potentiation of PPARγ-mediated transcription by troglitazone was eliminated by co-treatment with a PPARγ antagonist, SR-202 (Figure 3C), but the anti-HBV activity of troglitazone was retained even in the presence of SR-202 (Figure 3E). These results clearly indicated that PPARγ activity is not responsible for the anti-HBV activity of troglitazone.

### Troglitazone Inhibits HBV Internalization

We next investigated which step in the HBV life cycle was blocked by troglitazone. The HBV life cycle can be divided into two major phases: (1) the early phase of infection, including attachment, internalization, nuclear import, and cccDNA formation; and (2) the subsequent replication phase, including transcription, encapsidation, reverse transcription, and viral release (Figure 4A; Watashi et al., 2014). As the first experimental approach, we examined the effect of troglitazone on the HBV replication phase using HepG2.2.15.7 cells, which stably carry an HBV-encoding transgene and replicate HBV, but do not support the entry phase because of a deficiency for the NTCP entry receptor (Ogura et al., 2014; Iwamoto et al., 2017). HepG2.2.15.7 cells were treated with or without compounds for 6 days, and HBV DNA secreted into the culture supernatant was detected to evaluate HBV replication. Whereas, treatment with a positive control (entecavir, a clinically available nucleoside analog) yielded a decreased level of HBV DNA, troglitazone had no significant effect on HBV replication (Figure 4B). As a second step, we examined the effect of troglitazone on other hepatitis viruses, including hepatitis C virus (HCV) and hepatitis D virus (HDV). HDV possesses the same envelope as HBV and thus follows an entry process identical or very similar to that used by HBV, while hepatitis C virus (HCV) entry proceeds in an entirely different manner (Lindenbach and Rice, 2013). As shown in Figures 4C,D, troglitazone significantly inhibited infection by HDV, but not that by HCV, suggesting that troglitazone specifically blocked NTCP-mediated viral entry. As a third step, we examined whether troglitazone inhibited HBV attachment or internalization. To evaluate the effect of troglitazone on HBV attachment to the host cell surface, we used a fluorescently labeled myristoylated-preS1 peptide (preS1 probe) consisting of aa 2–48 of the preS1 region of the large HBs antigen; this region is essential for the binding of HBs to NTCP at the cell surface. As shown in Figure 4E, treatment with troglitazone did not have a clear effect to reduce preS1 attachment to the cells, while a preS1 peptide lacking the fluorescent label competitively decreased preS1-cell attachment (Figure 4E). To evaluate HBV internalization, HepG2-hNTCP-C4 cells were inoculated with HBV at 4°C (to allow cellular attachment) and then washed before culturing at 37°C for 16 h (to induce virus internalization into the cells) in either the presence or absence of compounds. HBV internalization was evaluated by quantifying intracellular HBV DNA after trypsin digestion (to remove any HBV remaining on the cell surface; Figure 4Fa; Watashi et al., 2014). The results showed that treatment with troglitazone reduced the level of internalized HBV DNA (Figure 4Fb). We further confirmed the preS1-mediated HBV internalization by imaging the localization of the fluorescently labeled preS1 probe, as reported for analyzing preS1-mediated internalization (König et al., 2014). HepG2-hNTCP-C4 cells were incubated with the preS1 probe at 37°C in the presence or absence of compounds (Figure 4Fc). After 8 h of incubation, the preS1 probe was observed to form punctate structures within the cells treated with DMSO (control) or with pioglitazone (Figure 4Fd, 2 and 8), which had no effect on HBV infection used as a negative control. However, most of the preS1 probe remained on the cell surface following treatment with troglitazone (Figure 4Fd, 5), consistent with the results of the internalization assay using the HBV inoculum (Figure 4Fb). Treatment with troglitazone did not affect the expression level of NTCP (Figure S2). Interestingly, while NTCP was internalized inside the cells in colocalization with preS1 without compound treatment (Figure 4Fd, 1 and 3), this NTCP internalization with preS1 was abrogated by treatment with troglitazone (Figure 4Fd, 4 and 6).

### Identification of Peptides That Interact With NTCP and Block NTCP Oligomerization

Many transporters [such as EAAT2 (excitatory amino acid transporter; SLC1A2), NBCe1-A (Na^+^-${\text{hco}}_{3}^{-}$ co-transporter; SLC4A4), GAT1 (GABA transporter; SLC6A1), GlyT2 (glycine transporter; SLC6A5), ASBT (apical sodium dependent bile acid transporter; SLC10A2), hNKCC2 (Na^+^-K^+^-2Cl^−^ co-transporter; SLC12A1), and hNKCC1 (SLC12A2)] form dimers or oligomers at the plasma membrane (Moore-Hoon and Turner, 2000; Kramer et al., 2001; Scholze et al., 2002; Starremans, 2003; Bartholomäus et al., 2008; Kao et al., 2008; Gebhardt et al., 2010; Bijsmans et al., 2012; Chothe et al., 2018). Among these examples, the oligomerization of GAT1 and GlyT2 was shown to modulate their membrane trafficking and to change the subcellular localization of these proteins (Scholze et al., 2002; Bartholomäus et al., 2008). It has been also reported that the induction of oligomerization of DAT (dopamine transporter; SLC6A3) by a small molecule triggers endocytosis of this transporter (Sorkina et al., 2018). Indeed, NTCP has been reported to form dimers in the rat liver membranes and U2OS cells (Bijsmans et al., 2012). Although the biological significance of NTCP dimerization is not fully understood, we hypothesized that the dimerization/oligomerization of NTCP may affect the protein's subcellular localization. We therefore sought to address this point using the following experimental approach.

We examined NTCP oligomerization by employing the proximity ligation assay (PLA), which can detect protein-protein interaction within cells, while also tracking NTCP's localization by use of a fluorescent tag. In HepG2 cells transiently expressing both myc-tagged (myc-NTCP) and HA-tagged (HA-NTCP) NTCP, PLA signal produced by myc-HA proximity, though low, exhibited a punctate distribution, including presence on the plasma membrane (Figures 5Aa–c). This pattern was consistent with the results of a previous paper showing NTCP dimerization on the cell surface (Bijsmans et al., 2012). There has been no report so far showing the impact of HBV infection on NTCP oligomerization. Interestingly, the observed level of fluorescence was clearly increased upon incubation with preS1 peptide, without affecting the expression level of NTCP (Figure S3), and the majority of the resulting PLA signal was colocalized with the preS1 probe (Figures 5Ad–f). These results suggest that NTCP oligomerization was promoted in the presence of preS1. Next, we used an *in vitro* pull-down assay to explore the regions in NTCP that are responsible for oligomerization. As binding counterparts of recombinant His-NTCP, we used a library of 34 synthetic biotinylated-NTCP fragment peptides consisting of 20-aa lengths of NTCP that together spanned the entire NTCP sequence (Figure 5B). His-NTCP was incubated with these biotinylated-NTCP peptides, -preS1 peptide (as a positive control), or -HCV E1 peptide (aa 111–140; as a non-relevant negative control) that had been immobilized on streptavidin-agarose beads; His-NTCP that coprecipitated with these peptides then was detected by immunoblotting with anti-His antibody. As shown in Figure 5B, His-NTCP coprecipitated with the preS1 peptide, but not with the HCV E1 peptide nor with agarose beads alone [“no-peptide”] (Figure 5Ba). The intensity of the His-NTCP band that coprecipitated with the NTCP fragment peptides was quantified by densitometry and is shown as a fold-change normalized to the binding obtained with the preS1 peptide (positive control; defined as 1.0 in Figure 5Bb). Among the peptides, two peptides—those consisting of aa 221–240 and aa 271–290 of NTCP—exhibited binding to His-NTCP at levels similar to or exceeding that obtained with the reference preS1 peptide (Figures 5Ba,b). Interaction of the NTCP(221–240) and NTCP(271–290) peptides with His-NTCP was also confirmed by surface plasmon resonance (SPR) analysis: NTCP peptides spanning aa 221–240 and aa 271–290, but not that spanning aa 1–20, yielded nominally dose-dependent SPR responses on a NTCP peptide-immobilized chip (Figure S4). These data suggested that the regions represented by the NTCP(221–240) and NTCP(271–290) peptides are responsible for NTCP oligomerization. These peptides therefore were used as decoys in a PLA of the ability to block NTCP oligomerization. Specifically, addition of an excess amount of either NTCP(221–240) or NTCP(271–290) yielded a significant decrease in the PLA signal of myc-HA proximity produced in the presence of preS1 peptide (Figure 5Cb, 4 vs. 8 or 10, and **5Cc**). These results suggested that the NTCP(221–240) and NTCP(271–290) fragment peptides can be used as competitive blockers of NTCP oligomerization (Figure 5C).

### Troglitazone or Peptides That Cause Dissociation of NTCP Oligomerization Inhibits Viral Internalization

**Figure 6:**
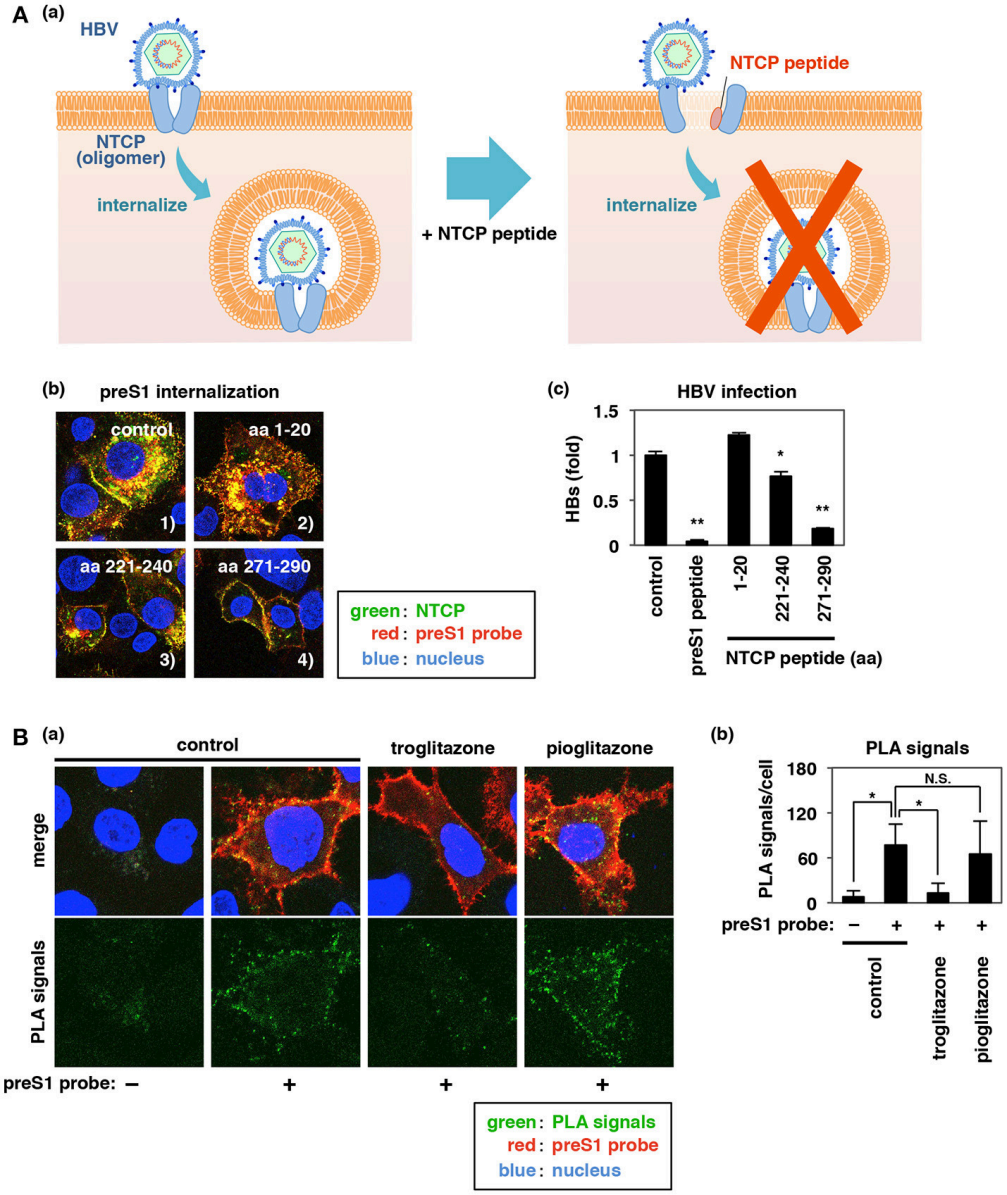
Inhibition of NTCP oligomerization by peptides or troglitazone impedes HBV preS1-NTCP internalization. (**Aa,b**) Effect of NTCP peptides (aa 1–20, aa 221–240, and aa 271–290) on preS1 internalization as described in Figures 4Dc,d. Merged patterns of green (NTCP), red (preS1 probe), and blue (nucleus) signals are shown. Intracellular dotted yellow signals (the internalized preS1-NTCP) were clearly observed in the control and with aa 1–20, while those signals were decreased with aa 221–240 and even more strongly decreased with aa 271–290. **(c)** HBV infection of HepG2-hNTCP-C4 cells was examined as described in Figure 1B following treatment with or without the indicated peptides [preS1 or NTCP peptides (aa 1–20, aa 221–240, or aa 271–290)] using detection of HBs antigen at 12 days post-infection. **(B)** Effect of troglitazone on NTCP oligomerization was evaluated by PLA, as shown in Figure 5A. PLA signal was detected in HepG2 cells overexpressing both myc-NTCP and HA-NTCP following treatment with or without preS1 probe in the presence or absence of troglitazone or pioglitazone. **(a)** The lower panels show the PLA signal (green) only, and the upper panels are the merged images of green (PLA signal), red (preS1 probe), and blue (nucleus). **(b)** PLA signals in the experiment in **(a)** were quantified using Dynamic Cell Count (KEYENCE). Signal was significantly reduced by troglitazone, but not by pioglitazone. Data are shown as mean ± SD. Statistical significance was determined using a two-tailed non-paired Student's *t*-test (N.S.; not significant, ^*^P < 0.05, ^**^P < 0.01).

To examine the role of NTCP oligomerization in HBV internalization, we evaluated preS1 internalization as well as HBV infection under competitive interference with NTCP oligomerization by the NTCP(221–240) or NTCP(271–290) peptides. As above (in Figure 4Fc), HepG2-hNTCP-C4 cells were exposed to preS1 probe; the treatment was then chased and subcellular localization of the probe (as well as the localization of NTCP) was monitored in both the presence and absence of excess amounts of NTCP(221–240) or NTCP(271–290; Figures 6Aa,b). After 8 h of preS1 incubation at 37°C, preS1 (red, Figure 6Ab) was observed as intracellular punctate structures that showed colocalization with NTCP (seen as yellow, Figure 6Ab) in the absence of the NTCP peptide or in the presence of NTCP(1–20; negative control). In contrast, decreased levels of internalized preS1 and NTCP were observed in the presence of NTCP(221–240) or NTCP(271–290), peptides shown to inhibit NTCP oligomerization (Figure 6Ab, panels 3 and 4). The inhibition of preS1-NTCP internalization by NTCP(271–290) was stronger than that by NTCP(221–240; Figure 6Ab, panel 4), consistent with the higher avidity of NTCP(271–290) for His-NTCP (Figure 5Bb). Furthermore, these two NTCP peptides, especially NTCP(271–290), significantly reduced the infection by HBV (Figure 6Ac) without any cytotoxicity (Figure S5). These results suggested that the agents that impede NTCP oligomerization also interfere with the internalization and infection of HBV. Consistent with this inference, we found that troglitazone significantly inhibited the PLA signal that was produced by NTCP oligomerization (Figure 6B). However, this inhibition of the PLA signal was not observed following treatment with pioglitazone (Figure 6B), an analog that lacks the ability to inhibit preS1 internalization (Figure 4F) and viral infection (Figure 2B). Although further analysis is needed to demonstrate the role of NTCP oligomerization in preS1-NTCP internalization, our study demonstrated that troglitazone inhibits HBV infection by interrupting viral internalization. Moreover, troglitazone and the peptides that block the NTCP oligomerization represent the first examples of inhibitors that target the cellular machinery for regulating HBV internalization.

### Discussion

In this study, a cell-based chemical screen identified troglitazone as a small molecule that inhibits HBV infection. As viral entry is essential for the initiation, spread, and maintenance of viral infection, this entry step is an attractive target for the development of antivirals. HBV entry inhibitors are expected to be useful for the prevention of vertical transmission and of HBV recurrence after liver transplantation, as well as for post-exposure prophylaxis. In addition, entry inhibitors are expected to have potential in multidrug treatments designed to improve the clinical outcome of anti-HBV treatment. So far, a series of HBV entry inhibitors have been identified that block viral attachment to the NTCP host receptor. Troglitazone differs from these previously identified entry inhibitors, representing a novel class of anti-HBV entry inhibitors that are suggested to interfere with the process of HBV internalization from the cell surface to an intracellular compartment. To date, viral internalization has been reported as a drug target in several viral systems, other than HBV. For instance, a flavor component of green tea, (–)-epigallocatechin-3-gallate, has been reported to inhibit influenza virus infection by targeting the viral membrane to block viral penetration of cells (Kim et al., 2013). Similarly, chloroquine, a clinically used anti-malarial agent, interferes with internalization of Zika and chikungunya viruses (Khan et al., 2010; Li et al., 2017). In another example, arbidol, an drug approved in Russia and China for the treatment of acute respiratory infections has been shown to inhibit HCV internalization (Blaising et al., 2013b). Other work showed that silibinin, an extract from the milk thistle plant *Silybum marianum*, exerts anti-HCV activity through inhibition of multiple steps of the HCV life cycle, including viral internalization, by affecting endosomal trafficking of virions (Wagoner et al., 2010, 2011; Dahari et al., 2011; Blaising et al., 2013a; Polyak et al., 2013). The clinical efficacies of silibinin in HCV-infected patients have been demonstrated in clinical trials with chronic hepatitis C patients and with individuals who had experienced graft reinfection following liver transplantation (Ferenci et al., 2008; Hawke et al., 2010; Beinhardt et al., 2011). Thus, viral internalization inhibitors are expected to contribute to the improved treatment as well as prevention of HBV infection. It is interesting that the cotreatment of troglitazone with myrcludex-B, an attachment inhibitor under clinical development (Blank et al., 2018), significantly increased the anti-HBV activity (Figure S6).

Many membrane transporters have been reported to be able to form oligomers, although the relevance of this function remains poorly understood. Notably, oligomerization has been shown to play a role in membrane trafficking, protein function, regulation, and/or turnover of membrane transporters, depending on the individual transport protein, including solute carrier (SLC) family transporter proteins such as EAAT2 (excitatory amino acid transporter; SLC1A2), NBCe1-A (Na^+^-${\text{HCO}}_{3}^{-}$ co-transporter; SLC4A4), GAT1 (GABA transporter; SLC6A1), GlyT2 (glycine transporter; SLC6A5), ASBT (apical sodium dependent bile acid transporter; SLC10A2), hNKCC2 (Na^+^-K^+^-2Cl^−^ co-transporter; SLC12A1), hNKCC1 (SLC12A2), and NTCP (SLC10A1) (Moore-Hoon and Turner, 2000; Kramer et al., 2001; Scholze et al., 2002; Starremans, 2003; Bartholomäus et al., 2008; Kao et al., 2008; Gebhardt et al., 2010; Bijsmans et al., 2012; Chothe et al., 2018). Among these examples, the oligomerization of GAT1 and GlyT2 has been suggested to regulate the trafficking of these transporters; specifically, oligomerization of these two proteins on the endoplasmic reticulum contributes to translocation to the plasma membrane (Scholze et al., 2002; Bartholomäus et al., 2008). Although the biological significance of NTCP oligomerization has not been well defined, our results suggest that the oligomerization of NTCP is significance for trafficking of this protein. Notably, interference with NTCP oligomerization (by either competitive peptides or troglitazone) significantly impaired the internalization of the NTCP-HBV preS1 complex and infection by HBV. Although further analysis will be needed to fully clarify the role of NTCP oligomerization and the mechanism whereby this process is regulated, the present study is significant in identifying peptides and small molecules that can be used as probes for analyzing these processes. In the present study, we used fluorescent-tag-labeled preS1 to visualize the internalization of the HBV envelope; this probe frequently has been used for the analysis of HBV entry (König et al., 2014). Future analysis of HBV internalization using labeled HBV particles also will be important, although labeling of HBV particles is currently difficult and so represents a technical challenge. However, the present study suggests that the oligomerization of NTCP can serve as a new target for the development of anti-HBV agents. Further optimization starting from troglitazone or the NTCP fragment peptides is expected to contribute to the development of such agents. Indeed, a novel thiazolidinedione, inolitazone, is under clinical development as an anticancer drug for treatment of anaplastic thyroid cancer and metastatic colorectal cancer (Smallridge et al., 2013; Komatsu et al., 2014; Murakami et al., 2014). Thus, compounds under clinical development or drugs that are already clinically available (for treatment of other diseases) are expected to facilitate the further development of this novel class of potential anti-HBV agents.

## Author Contributions

KW designed the study and critically revised the manuscript. KF, ST, and KW screened compounds in the cell-based screen. KF, ST, MOs, and KW performed biological experiments. KF, ST, and KW wrote the paper. KF, ST, MOs, RS, HA, MOh, S-YP, MM, TW, CS, YO, and KW analyzed and discussed the results. KW supervised the project.

### Conflict of Interest Statement

The authors declare that the research was conducted in the absence of any commercial or financial relationships that could be construed as a potential conflict of interest.
