# Supplementary material for: Troglitazone Impedes the Oligomerization of Sodium Taurocholate Cotransporting Polypeptide and Entry of Hepatitis B Virus Into Hepatocytes
Source: Front Microbiol. 2019 Jan 8;9:3257. doi: 10.3389/fmicb.2018.03257 (PMC6331526; doi:10.3389/fmicb.2018.03257)
Supplement: Supplementary file 1 [file Data_Sheet_1.docx]

Supplementary Material

Troglitazone impedes the oligomerization of sodium taurocholate cotransporting polypeptide and entry of hepatitis B virus into hepatocytes

Kento Fukano^1,2^, Senko Tsukuda^1,3^, Mizuki Oshima^1,4^, Ryosuke Suzuki^1^, Hideki Aizaki^1^, Mio Ohki^5^, Sam-Yong Park^5^, Masamichi Muramatsu^1^, Takaji Wakita^1^, Camille Sureau^6^, Yuki Ogasawara^2^, Koichi Watashi^1,4,7*^

^1^Department of Virology II, National Institute of Infectious Diseases, Tokyo, Japan

^2^Department of Analytical Biochemistry, Meiji Pharmaceutical University, Kiyose, Japan

^3^Liver Cancer Prevention Research Unit, Center for Integrative Medical Sciences, RIKEN, Wako, Japan

^4^Department of Applied Biological Science, Tokyo University of Science, Noda, Japan

^5^Drug Design Laboratory, Graduate School of Medical Life Science, Yokohama City University

^6^Laboratoire de Virologie Moléculaire, Institut National de la Transfusion Sanguine, CNRS, INSERM U1134, Paris, France

^7^JST CREST, Saitama, Japan

*** Correspondence:**Dr. Koichi Watashi
Department of Virology II, National Institute of Infectious Diseases, 1-23-1 Toyama, Shinjuku-ku, Tokyo 162-8640, Japan (E-mail: kwatashi@nih.go.jp)

**Keywords:** HBV, internalization, NTCP, oligomerization, multimerization, troglitazone, entry, preS1

# Supplementary Materials and Methods

**Surface plasmon resonance (SPR) analysis**

# Binding affinity between recombinant His-NTCP and NTCP peptides was evaluated by SPR analysis using a Biacore X100 (GE Healthcare) instrument as described previously (Kaneko et al., 2018). His-NTCP diluted to the indicated concentrations with running buffer (10 mM HEPES pH 7.4, 150 mM NaCl, 3 mM EDTA, 0.05% Tween 20, 5% DMSO) was injected for 120 seconds over NTCP peptides that had been immobilized on streptavidin-coated sensor chips. The resulting data were analyzed using Biacore X100 evaluation software (GE Healthcare, ver. 2.0.1).

**siRNA transfection**

siRNA was transfected to the cells at 30 nM using Lipofectamine RNAiMAX (Thermo Fisher Scientific) according to the manufacturer’s protocol.

# Supplementary Figures

# Supplementary Figure 1.

# Supplementary Figure 1. The raw data of the immunoblot shown in Fig. 5B-a.

**Supplementary Figure 2.**

**Supplementary Figure 2.** NTCP (upper panel) and actin as an internal control (lower panel) were examined by immunoblot analysis of primary human hepatocytes treated with or without the indicated compounds (40 μM Ro41-5253 or 25 μM troglitazone, pioglitazone, or ciglitazone) for 72 h.

**Supplementary Figure 3.**

**Supplementary Figure 3.** NTCP (upper panel) and actin as an internal control (lower panel) were examined by immunoblot analysis of HepG2-hNTCP-C4 cells transfected with 30 nM si-NTCP for 72 h or treated with or without 40, 80, and 160 nM preS1 peptide for 8 h. The values below the panels indicate the relative band intensities for these proteins quantified by densitometry.

**Supplementary Figure 4.**

**Supplementary Figure 4.** Surface plasmon resonance (SPR) analysis of interaction between recombinant His-NTCP and the NTCP fragment peptides (aa 1-20, aa 221-240, or aa 271-290). Varying concentrations (500, 250, 125, and 62.5 nM) of His-NTCP were incubated over a sensor chip immobilized with the indicated NTCP fragment peptides from 0 to 120 seconds. After 120 seconds, compound-free buffer was incubated. The SPR responses are indicated in resonance unit (RU).

**Supplementary Figure 5.**

**Supplementary Figure 5.** Cell viability of HepG2-hNTCP-C4 cells treated with or without the indicated peptides [preS1 or NTCP peptides (aa 1-20, aa 221-240, or aa 271-290)] was measured by MTT assay.

**Supplementary Figure 6.**

**Supplementary Figure 6.** Cotreatment of troglitazone with an attachment inhibitor, myrcludex-B. HepG2-hNTCP-C4 cells treated with the indicated concentrations and combination of compounds were inoculated with HBV as shown in Fig. 1B. HBV infection was evaluated by detecting HBs antigen secreted into the culture supernatant by ELISA. Data are shown as mean ± SD. Statistical significance was determined using a two-tailed non-paired Student’s t-test (**P* < 0.05, ***P* < 0.01).
